# Prosociality from early adolescence to young adulthood: A longitudinal study of individuals with a history of language impairment

**DOI:** 10.1016/j.ridd.2017.01.018

**Published:** 2017-03

**Authors:** Umar Toseeb, Andrew Pickles, Kevin Durkin, Nicola Botting, Gina Conti-Ramsden

**Affiliations:** a Department of Psychology, Manchester Metropolitan University, Brooks Building, 53 Bonsall Street, Manchester M15 6GX, UK; b Department of Biostatistics, Institute of Psychiatry, King’s College London, De Crespigny Park, Denmark Hill, London, SE5 8AF, UK; c School of Psychological Sciences and Health, University of Strathclyde, 40 George Street, Glasgow, G1 1QE, UK; d Language and Communication Science, Northampton Square, City University, London, EC1 V0HB, UK; e School of Health Sciences, The University of Manchester, Ellen Wilkinson Building, Oxford Road, Manchester M13 9PL, UK

**Keywords:** Prosociality, Language impairment, SDQ, Longitudinal, Early adolescence, Young adulthood

## Abstract

**Background:**

Longitudinal research into the development of prosociality contributes vitally to understanding of individual differences in psychosocial outcomes. Most of the research to date has been concerned with prosocial behaviour in typically developing young people; much less has been directed to the course of development in individuals with developmental disorders.

**Aims:**

This study reports a longitudinal investigation of prosocial behaviour in young people with language impairment (LI), and compares trajectories of development to typically developing age-matched peers (AMPs).

**Methods and procedures:**

Participants were followed from age 11 years to young adulthood (age 24 years).

**Outcomes and results:**

Participants with LI perceived themselves as prosocial; their ratings – though lower than those for the AMPs – were well within the normal range and they remained consistently so from 11 to 24 years. Two different developmental trajectories were identified for the LI group, which were stable and differed only in level of prosociality. Approximately one third of participants with LI followed a moderate prosociality trajectory whilst the majority (71%) followed a prosocial trajectory. We found evidence of protective effects of prosociality for social outcomes in young adulthood.

**Conclusions and implications:**

The findings indicate that prosociality is an area of relative strength in LI.

**What this paper adds?:**

To our knowledge, this is the first study to examine developmental changes in levels of prosociality from early adolescence to young adulthood in a cohort of young people with LI. Approximately one third of participants with LI followed a moderate prosociality trajectory whilst the majority (71%) followed a prosocial trajectory. We argue that prosociality is different to other areas of functioning in LI. Prosociality appears to be an area of relative strength and can act as a protective factor in social functioning. Prosociality was associated with better community integration in young adulthood and was significantly protective against friendship difficulties for individuals with LI. This paper also raises the thought-provoking issue of potential distal effects of early identification and intensive support for LI. It is important to note that all of the participants with LI in this study had been identified as having language difficulties in childhood and had received intensive intervention for their difficulties in language units attached to mainstream schools across England. The early identification of language difficulties and the context of early, intensive language support received in educational contexts such as language units may have nurtured socialisation processes and the development of emphatic concern, which in turn influence the development of prosociality later in young adulthood. More individual differences in prosociality have been reported for other samples drawn from a variety of schools with different educational provision and levels of language support and younger age groups, such as primary school-aged children with LI.

## Highlights

• This is the first longitudinal study of prosociality in young adults with LI.
• Participants with LI perceived themselves as prosocial.
• Ratings remained within the expected range across young adulthood (11–24 years).
• Two different developmental trajectories were identified for the LI group.
• Small to medium effects were found indicating protective effects of prosociality into adulthood.

## 1 Introduction

Prosociality involves behaviours that are positively responsive to others’ needs and welfare. Examples include being helpful and sharing, showing kindness and consideration, cooperating with others and expressing empathy and sympathy. Why and how prosociality develops is not fully understood but theories and evidence point to a multifactorial process, involving guidance from socialisation agents (such as modelling and reinforcement by parents or teachers, learning social and moral norms), genetic heritability, and emotional and social-cognitive development (Eisenberg, Fabes, & Spinrad, 2006; Jensen, Vaish, & Schmidt, 2014). Most of the research to date has been concerned with prosocial behaviour in typically developing young people; much less has been directed to the course of development in individuals with developmental disorders. Young people with disorders are at greater risk of social exclusion and so the extent to which they do manifest prosocial behaviours is an important question, with implications for our theoretical accounts of what factors influence progress in this domain and our understanding of what influences wellbeing in those with disabilities. In the present paper, we report a longitudinal investigation of prosocial behaviour in young people with language impairment (LI), followed through adolescence into early adulthood.

### 1.1 Prosociality: developmental change and individual differences

Given that multiple factors bear on prosociality, it is to be expected that prosocial behaviour will be subject to both developmental changes and individual differences. Prosocial behaviours are evident from infancy (Liszkowski, Carpenter, & Tomasello, 2008; Warneken & Tomasello, 2007) but they become more elaborate − and more nuanced − with development and, at any age, some individuals exhibit them more than others (Eisenberg & Fabes, 1998).

From the toddler years through early childhood, children tend to show an increase in the frequency of prosocial behaviours (Eisenberg & Fabes, 1998). Through middle childhood, the findings are more mixed, with some studies suggesting stability (Cote, Tremblay, Nagin, Zoccolillo, & Vitaro, 2002; Flynn, Ehrenreich, Beron, & Underwood, 2015) but others finding modest declines (Kokko, Tremblay, Lacourse, Nagin, & Vitaro, 2006). During adolescence, some evidence points to a gradual decline in prosocial behaviours but with a possible rebound in late adolescence/early adulthood (Carlo, Crockett, Randall, & Roesch, 2007; Kanacri, Pastorelli, Eisenberg, Zuffiano, & Caprara, 2013; Spinrad & Eisenberg, 2009). At all of these stages, the overall picture is qualified by considerations including the beneficiaries of the behaviour, normative and situational variables − and individual differences, with different groups of individuals manifesting different trajectories (Nantel-Vivier et al., 2009). Within individuals, research by Eisenberg and colleagues on developmental trajectories has revealed significant, albeit modest, rank-order consistency in prosocial behaviours over time and contexts from the preschool years to early adulthood (Eisenberg, Miller, Shell, McNalley, & Shea, 1991; Eisenberg et al., 2002).

Longitudinal studies of development from adolescence to adulthood remain sparse. Three main trajectory groups have been identified: prosocial (and increasing from adolescence 16/17 years to young adulthood 22/23 years), moderate prosocial, and low prosocial; the latter two groups having stable trajectories from adolescence to early adulthood (Kanacri, Pastorelli, Zuffiano et al., 2014). In order to distinguish the three trajectories found, Kanacri et al. refer to the prosocial trajectory as “high” prosocial (in relation to what they refer to as moderate and low). However, it is important to note that the scores for the participants they refer to as “high” prosocial are close to the average of the 1–9 point scale they used.

Analyses from the same research group working with a large cohort of Italian children have revealed more variability when trajectories are modelled from early adolescence (age 13 years) to young adulthood (Kanacri, Pastorelli, Eisenberg et al., 2014). Taken together, findings suggest that individuals may show some fluctuations in prosocial development from childhood to young adulthood though radical shifts (e.g., from being low prosocial to becoming prosocial) are not common.

Gender differences in prosociality have been consistently observed. Generally, girls score more highly than boys on measures of prosociality (Kanacri et al., 2013) and boys are less likely to follow a high prosociality trajectory (Nantel-Vivier, Pihl, Cote, & Tremblay, 2014).

### 1.2 Prosocial behaviours: positive and protective?

Prosocial behaviours are conducive to positive social relations. Prosocial children are more accepted and more popular among their peers (Asher & Coie, 1990; Zimmer-Gembeck, Geiger, & Crick, 2005). In adolescence, prosociality is associated with social bonding and favourable friendship qualities (Cillessen, Jiang, West, & Laszkowski, 2005; Markiewcz, Doyle, & Brendgen, 2001). Prosocial behaviour in young adulthood has been found to be associated with greater involvement in the community (Kanacri, Pastorelli, Zuffiano et al., 2014).

As well as contributing to positive social relationships, there is accumulating evidence that prosocial attributes and experiences may mitigate the effects of some factors that place young people at risk of adverse outcomes. Prosocial adolescents have been reported to be less likely to manifest antisocial and delinquent behaviour (Carlo et al., 2014; Pursell, Laursen, Rubin, Booth-LaForce, & Rose-Krasnor, 2008). Participation in prosocial peer relationships appears to provide support for children who have negative experiences (such as victimisation), facilitating coping and psychosocial resilience (Griese & Buhs, 2014; Martin & Huebner, 2007).

### 1.3 Prosociality and language abilities

Many factors are involved in the development of prosociality, and some of these are discussed in a large research literature (Eisenberg & Fabes, 1998; Eisenberg, Cumberland, Guthrie, Murphy, & Shepard, 2005; Eisenberg, Fabes, Spinrad, & 2006). However, an ability that may contribute to initiating and managing prosocial behaviours has received scant attention: language. Relatively little research has addressed the extent to which language ability bears on prosociality in children and young people. Yet language is the primary medium through which human beings communicate. It is possible to offer help to others, to share material possessions or emotions, to show kindness and consideration, to express empathy and sympathy without using language − but the likelihood is that most of these, and other, prosocial activities will involve speaking and listening, as do most human interactions from childhood through adolescence and beyond.

Within this context, individuals with language impairment (LI) are of particular interest. How do they fare in prosocial skills, if they have deficits in expressing themselves and comprehending the subtleties of others’ language?

Language impairment affects approximately 7% of children at school entry (Tomblin et al., 1997). Children with LI have problems putting words together (expressive language) and/or understanding what others say to them (receptive language) in the absence of learning difficulties or sensory problems such as deafness. There has been and continues to be much debate about the diagnostic criteria and terminology to describe the difficulties experienced by children and young people with LI (Bishop, 2014, Reilly et al., 2014). There is consensus however, that although LI is characterised by language difficulties during childhood, the disorder often persist into adolescence and young adulthood. There is also consensus that LI is heterogeneous and can be associated with difficulties beyond language. For example, motor functioning (Finlay & McPhillips, 2013) and memory abilities (Lum, Conti-Ramsden, Page, & Ullman, 2012).

The few studies involving prosociality in children with LI have been mainly cross-sectional in design, have involved relatively small numbers of participants, and the findings have been mixed. For example, it has been found that children with LI attending primary school are rated by their teachers as being less prosocial and more prone to withdrawal than their peers. Nonetheless, overall levels of prosociality are not in the abnormal range (Brinton, Fujiki, Montague, & Hanton, 2000; Fujiki, Brinton, Morgan, & Hart, 1999; Hart, Fujiki, Brinton, & Hart, 2004), and standard deviations suggest large individual differences (Bakopoulou & Dockrell, 2016). The one longitudinal study of prosocial behaviours in children with LI (Lindsay & Dockrell, 2012) followed 65 children from 8 to 16 years, and examined prosocial behaviours using teacher report. On average, children with LI scored within the normal range, but there were individual differences. The children in this study exhibited stable trajectories, with a rise in prosociality evident between the ages of 8–12 years.

Thus, the picture emerging to date shows that individuals with LI can certainly participate prosocially though, overall, they may do so less skilfully and less successfully than children without LI. Lindsay and Dockrell’s (2012) findings indicate increases in prosocial behaviour in those with LI in late childhood, which could reflect general developmental progress and/or gradual improvements in language abilities. Nevertheless, the amount of evidence available is small and only one study has addressed longitudinal trajectories in this population. Research on the associations between level of prosociality and outcomes in individuals with LI in young adulthood, has been scant (Brinton and Fujiki, 1999, Durkin and Conti-Ramsden, 2007, Durkin and Conti-Ramsden, 2010; Mok, Pickles, Durkin, & Conti-Ramsden, 2014). In particular, an important question remains unanswered: Does prosociality confer protection against other developmental risks in the face of LI?

### 1.4 The present study: questions and hypotheses

In this investigation we examine longitudinal development of prosociality from early adolescence (age 11 years) to young adulthood (age 24 years) in young people with and without a history of LI. The study was motivated by three main questions: Do adolescents and young adults with LI differ in prosocial orientation to age-matched, typically developing peers (AMPs)? Do those with LI show similar developmental trajectories to typically developing youth? And is there any evidence that being prosocial provides a protective factor, associated with more positive outcomes on other measures of social and behavioural functioning?

With respect to differences between the groups in overall prosociality, the limited evidence available from studies earlier in development led us to expect that, on average, the LI group’s prosocial scores should fall in the normal range but somewhat lower than those of the AMP group (Lindsay & Dockrell, 2012). This would reflect the facts that individuals with LI have greater difficulties in participating in social life, tend to be less likely to initiate interactions, and have a lower sense of independence than AMPs (Brinton & Fujiki, 1999; Brinton, Spackman, Fujiki, & Ricks, 2007; Conti-Ramsden & Durkin, 2008; Durkin & Conti-Ramsden, 2010). These handicaps present impediments (though not necessarily insuperable barriers) to positive social interactions and helpfulness. An alternative hypothesis which should be acknowledged is that, as it is possible to behave prosocially with relatively little language (as demonstrated by infants and toddlers), it could be that those with LI could have adapted to their impairments by finding other ways of demonstrating prosociality.

Whether young people with LI show similar or different trajectory patterns to those of AMPs remains an empirical question. In terms of their language development, those with LI continue to develop their language skills into adolescence (Conti-Ramsden, St. Clair, Pickles, & Durkin, 2012) and they follow similar language trajectories to AMPs, but with a lag (Rice, 2004). As developmental language problems tend to impact on many other aspects of development, it could be that patterns of prosocial development in this group would be similar to those of AMPs, but with the timing of any accelerations or declines delayed. Alternatively, it is possible that being ‘out of synch’ with the communicative skills development of the majority of one’s peers puts an individual at risk of lower engagement in social activity and hence affords less opportunity to develop prosocial skills.

Finally, we examined whether different trajectories of prosociality were more or less protective of behavioural and social difficulties. Specifically, we examined friendship difficulties, community integration, aggressive behaviour and rule breaking. We predicted that having higher prosocial skills should be associated with more favourable outcomes in early adulthood in both LI and AMP groups.

## 2 Methods

### 2.1 Ethics

The study reported here received ethical approval from The University of Manchester.

### 2.2 Participants

#### 2.2.1 Participants with LI

Participants with LI (used throughout for ease) had a history of LI and were part of the [*name of study and references removed for blind-review*]. The initial cohort of 242 children, which consisted of 186 boys (77%) and 56 girls (23%), were recruited from 118 language units across England and represented a random sample of 50% of all 7-year olds attending language units for at least half of the school week. Language units are specialised classes for children who have been identified with primary language difficulties. Individuals were contacted again at ages 8 (*n* = 232), 11 (*n* = 200), 14 (*n* = 113), 16 (*n* = 139), 17 (*n* = 85), and 24 (*n* = 84). The attrition observed was partly due to funding constraints at follow-up stages of the study. The sample of participants with LI did not differ between baseline and each of the follow up stages in standard scores of: age 11(receptive language (*t*(240) = 0.42, *p* = .676), expressive language (*t*(229) = 1.79, *p* = .076), or nonverbal IQ (*t*(231) = −.01, *p* = .991)), age 16 (receptive language (*t*(240) = −0.865, *p* = .388), expressive language (*t*(229) = −.64, *p* = .521), or nonverbal IQ (*t*(231) = −.188, *p* = .851)), and age 24 (receptive language (*t*(240) = −1.13, *p* = .261), expressive language (*t*(229) = −.45, *p* = .634), or nonverbal IQ (*t*(231) = −.60, *p* = .545)).

Prosociality was ascertained at ages 11, 16, and 24 years. Thus, for the current investigation, analyses were undertaken for three time points only. These are referred to as time 1 (T1), time 2 (T2), and time 3 (T3). Participants were included in the analyses if data were available at least 2 of the 3 time points. At T1 (mean age 10 years 11 months, SD 5 months) and T2 (mean age 15 years 10 months, SD 5 months), there were 130 participants (92 male and 38 female). At T3 (mean age 24 years 5 months, SD 9 months) there were 84 participants (56 male and 28 female). There were 73 LI participants who provided data at all three time points.

#### 2.2.2 Age-matched peers (AMP)

The comparison sample consisted of 65 AMPs (38 male and 27 female) and provided data at both T2 (mean age 15 years 11 months, SD 5 months) and T3 (mean age 23 years 11 months, SD 10 months). The comparison group of peers was selected to be of similar age, similar geographical area, and similar socioeconomic background as the young people with LI. The comparison group of AMPs were of a similar age to the sample with LI at each time point (T2: M 16.4, SD 0.4 years, T3: M 24.1, SD 0.9 years). AMP participants at age 16 (T2) came from similar geographical locations as the sample with LI. AMPs came from the same schools as the participants with LI as well as additional targeted schools to ensure a similar urban versus rural geographical distribution in both groups. In addition, participants in the AMP comparison group were sampled from selected demographic areas in order to ensure comparison peers came from a broad range of socioeconomic backgrounds, similar to participants with a history of LI. The LI and the comparison groups did not differ on household income at age 16years, T2 (χ^2^(10, N = 145) = 9.32, *p* = .501) nor personal income at age 24 years, T3 (χ^2^(5, N = 131) = 7.38, *p* = .194). AMPs had no history of special educational needs or speech and language therapy provision. At T2, 124 AMPs (76 males and 48 females) were recruited. Of these, 65 AMPs continued to participate at T3. Those who continued to participate at T3 had higher receptive language abilities (t(122) = 3.91, p < .001 95% CI [4.32, 13.2]) and PIQ scores (t(122) = 3.09, *p* = .002 95%CI [3.04, 13.92]) than those who did not. There were, however, no differences in gender (χ^2^(1, N = 124) = 0.46, *p* = .497) or expressive language abilities (t(122) = 1.34, *p* = .183 95% CI [−1.71, 8.92]) between those who participated at T3 and those who did not. The psycholinguistic profiles of the participants are shown in Table 1.

### 2.3 Measures

#### 2.3.1 Language and nonverbal IQ

The Recalling Sentences subtest of the Clinical Evaluation of Language Fundamentals was used to assess expressive language (CELF-R, Semel, Wiig, & Secord, 1987; CELF-IV, Semel, Wiig, & Secord, 2003). At T1, the Test for Reception of Grammar (Bishop, 1982) was used to assess receptive language. The Word Classes subtest of the CELF was used to assess receptive language at T2 (CELF-R) and T3 (CELF-IV). Nonverbal IQ was measured at T1 and T2 using the Wechsler Intelligence Scale for Children Third Edition (WISC-III UK, Wechsler, 1992) and at T3 using the Wechsler Abbreviated Scale of Intelligence (Wechsler, 1999).

#### 2.3.2 Prosocial behaviour

The prosocial subscale of the Strengths and Difficulties Questionnaire (SDQ) (Goodman, 1997) was completed by the participants (self-report) at all three time points. The scale has good internal reliability (Goodman, Meltzer, & Bailey, 1998). The scale consists of 5 items each being coded as 0 = *Not true*, 1 = *Somewhat true*, and 2 = *Certainly true*. The items were: “I try to be nice to other people”, “I usually share with others”, “I am helpful if someone is hurt, upset or feeling ill”, “I am kind to younger children”, and “I often volunteer to help others”. Sum scores for the subscale range from 0 to 10 and for self-report are categorised as “Normal” (6–10), “Borderline” (5), and “Abnormal” (0–4). In a population sample of adolescents (Goodman, Lamping, & Ploubidis, 2010), the construct validity of the SDQ was shown to be at an acceptable level (factor loadings 0.56-0.76). Agreement between parent report and self-report was modest (0.34) and test-retest correlations were good (0.62) (Goodman, 2001). The internal reliability of prosocial subscale of the SDQ in the sample was good (Cronbach’s α = .71). This was comparable to the internal reliability of the subscale in population samples of young people (Cronbach’s α = 0.64–0.72, Giannakopoulos et al., 2009; Van Roy, Groholt, Heyerdahl, & Clench-Aas, 2006). The prosocial subscale is positively skewed in the general population of young people (M 8.0, SD 1.7, Meltzer, Gatward, Goodman, & Ford, 2000). This is in contrast to the other subscales of the SDQ, which measure difficulties, and so are negatively skewed (e.g. emotional difficulties M 2.8 SD 2.1). In addition, we examined stability of the prosocial subscale across time. An exploratory factor analysis was run for each of the three time points using the five items on the SDQ prosocial subscale. Inspecting the scree plots and the eigenvalues determined the number of factors. The five items loaded onto a single factor with high eigenvalues at each of the time points (T1 = 2.34, T2 = 2.04, T3 = 2.34), suggesting stability of the prosocial scores across time

#### 2.3.3 Friendship difficulties

At T3, a Friendship Difficulty Index (FDI) was created based on the Social Emotional Functioning Interview (SEF-I, Mawhood, Howlin, & Rutter, 2000). Participants were asked questions about their perception of acquaintances (range 0–2), description of current friendships (range 0–3), and their concept of friendship (range 0–3). Scores from the 3 questions were summed to create a total score (range 0–8). Higher summed scores indicated more friendship difficulties. The reliability of FDI in the sample was very good (Cronbach’s α = .84).

#### 2.3.4 Community integration

At T3, the Community Integration Measure (CIM, McColl, Davies, Carlson, Johnston, & Minnes, 2001) was used. The 10-item checklist (e.g., I feel like part of this community, like I belong here) were scored on a 5-point Likert scale: 1 “*Always disagree*”, 2 “*Sometimes disagree*”, 3 “*Neutral*”, 4 “*Sometimes agree*”, 5 “*Always agree*”. Higher summed scores represent a higher level of community integration. The reliability of the CIM in the sample was very good (Cronbach’s α = .83).

#### 2.3.5 Aggressive and rule breaking behaviour

At T3, two subscales of the Achenbach Checklist (Achenbach, 1991) were used: Aggressive Behaviour (15 items) e.g. “I argue a lot” and Rule Breaking (14 items) e.g. “I don't feel guilty after doing something I shouldn't”. All items were scored as 0 “*Not true*”, 1 “*Somewhat or sometimes true*”, or 2 “*Very true or very often*”. Higher summed scores indicated more difficulties. For both the aggressive behaviour (Cronbach α = .86) and rule breaking (Cronbach α = .72) the reliability of the both subscales was good.

### 2.4 Informed consent

The study reported here received ethical approval from The University of Manchester Research Ethics Committee, UK. Informed consent was obtained from all individual participants included in the study. Parents or legal guardians provided informed consent for all participants up to the age of 16 years. Participants themselves were asked if they wished to take part (at all phases) and provided written informed consent at ages 16 and 24 years.

### 2.5 Procedure

The participants were interviewed face-to-face at school or at their home on the measures described above as part of a wider battery. Interviews took place in a quiet room, wherever possible with only the participant and a trained researcher present. Standardised assessments of nonverbal and verbal skills were administered in the manner specified by the test manuals. During the interview, the items were read aloud to the participants. The items and response options were also presented visually to ensure comprehension. The authors complied with APA ethical standards in the treatment of the sample.

### 2.6 Latent class analysis

All statistical analyses were conducted using Stata/SE 13.1 (StataCorp, 2013). The ‘gllamm’ (generalized linear latent and mixed models; www. gllamm.org; Rabe-Hesketh, Skrondal, & Pickles, 2004) procedure command was used to model the changes in self-report prosocial scores across time. Latent classes (or groups) of individuals with similar patterns over time (Nagin & Odgers, 2010; Pickles & Davies, 1985) were identified using ordinal logistic models. Although the scale ranged from 0 to 10, there were only a small number of individuals who scored 0 or 1 (n = 3). Therefore, a score of 0 or 1 was recoded as 2. In doing this, the scale ranged from 2 to 10. The data was treated as missing at random. The gllamm command, which was used to for the latent class analysis, makes use of Maximum Likelihood Estimation to estimate model parameters. Intercept only, linear, and quadratic models were run with an increasing number of groups. The model used for further analyses was selected using both statistical goodness-of-fit criteria and interpretability. The Akaike information criterion (AIC) and Bayesian information criterion (BIC), which penalises more complex models, were used to assess the model fit. The most parsimonious model was the one with the lowest criterion value (Pickles & Croudace, 2010). The chosen model was then used to calculate for each participant the empirical Bayes’ estimates for the posterior probability of belonging to each trajectory group, and each participant was assigned to the trajectory group with the highest posterior probability. In addition, given the developmental period examined in this study (from childhood to young adulthood) and our aim to investigate mean-level differences over time, it was deemed necessary to test for scalar invariance of the SDQ prosocial subscale. We thus re-ran the above analysis using the gllamm command, and included link option (ologit) for conditional densities. Multiple links were specified using the lv option (time). The model still yielded a 2 class solution as the best solution, which suggests scale invariance can be assumed in the interpretation of the findings.

## 3 Results

### 3.1 Level of prosocial functioning

Both groups of participants reported prosocial behaviours within the normal range (clinical cut-off ≤ 4, Goodman, 1997). Mean prosocial scores for participants with LI were 8.0 (SD 2.2), 7.8 (SD 1.9) and 7.9 (SD 1.9) at T1, T2, and T3, respectively and for AMP mean scores were 8.8 (SD 1.3) and 8.6 (SD 1.5) at T2 and T3. In each group, only a minority of individuals (between 2 and 6%) reported levels of prosociality in the abnormal range at one time point. There were no individuals in either the LI or the AMP group who scored consistently low, in the abnormal range, during the timeframe studied. Prosocial scores were submitted to a 2 (Group: LI or AMP) x 2 (Time: T2 & T3) mixed ANOVA, with repeated measures on the latter factor. This analysis yielded a significant main effect of group, *F*(1,336) = 14.0, *p* <. 0001, *η^2^* = .04, but there was no main effect of time, *F*(1, 336) = .02, *p* = .90, nor an interaction between the two, *F*(1, 336) = .26, *p* = .61. Given the main effect of group, we undertook latent class analysis for LI and AMP separately.

### 3.2 Trajectories of prosociality from early adolescence to young adulthood

**Fig. 1:**
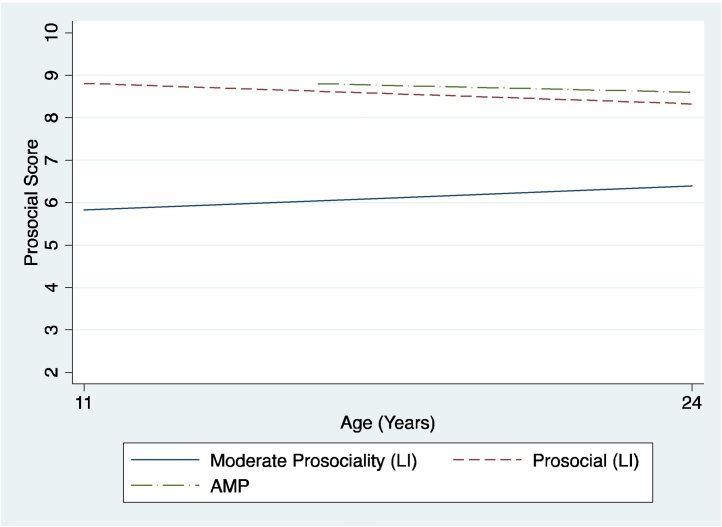
Trajectories of prosociality.

Intercept only, linear, and quadratic models were run with increasing numbers of classes starting with 1 class. For individuals with LI, the most parsimonious model was the intercept only 2-class solution. For the AMPs, it was the intercept only 1-class solution. The model fit statistics are shown in Table 2 and the trajectories are presented in Fig. 1. To aid with the understanding of Fig. 1, mean prosocial scores are presented in Table 3, which demonstrate the stability of prosociality over time.

The two distinct LI trajectory classes (for ease “trajectory” henceforth) had mean scores of 8.6 (1.4) and 6.0 (1.8) respectively. Given that the population mean for the SDQ prosocial subscale for 5–15 year olds is 8.0 (1.7) (Meltzer et al., 2000), we refer to these classes as prosocial and moderate prosociality respectively. Seventy one percent of LI participants (*n* = 93) were classified as following a prosocial trajectory and 29% of LI participants (*n* = 38) were classified as following a moderate prosociality trajectory. There was a significantly larger proportion of females in the prosocial trajectory (89.5% of females vs 63.4% of males, (*χ^2^*(1, N = 131) = 8.88, *p* = 003). Age-matched peers all followed a prosocial trajectory with mean scores of 8.7(SD 1.4).

It is known that the number of trajectory classes identified can depend upon the number of measurement occasions available (Lindsay, Clogg, & Grego, 1991). To investigate this potential effect further, models were fitted combining the LI and AMP participants into a single sample. The results were very similar to the findings examining LI and AMP samples separately. The best fitting model was a two-group intercept only model (prosocial and moderate prosociality) with a comparable number of LI participants in both groups as found with the LI sample only models. The majority of AMP participants were classified as following a prosocial trajectory. There were only 4 AMP participants following a moderate prosociality trajectory.

### 3.3 Outcomes at age 24

A number of one-way ANOVAs were run to investigate differences between the three prosociality groups (LI Moderate Prosociality, LI Prosocial, & AMP) for outcomes at age 24 years (see Table 4). Post hoc comparisons between the prosocial vs moderate prosociality LI groups revealed that being in the LI prosocial trajectory was significantly protective in the social domain, specifically friendship difficulties and community integration. No significant differences between the prosocial vs moderate prosociality LI groups were observed in the behavioural domains as measured by the Achenbach subscales on aggression and rule-breaking. Comparisons between LI groups with AMP revealed some significant differences in social and behavioural domains. The correlations between language, PIQ and outcomes at age 24 (T3) for study participants can be found in the Appendix A.

## 4 Discussion

### 4.1 Language and prosociality: young people with LI are prosocial

Participants with LI perceived themselves as prosocial; their ratings were well-within the normal range and they remained consistently so from 11 to 24 years. Mean prosocial scores for the group with LI were lower than those of their AMPs but still in the positive range according to SDQ norms. A history of language difficulties does not therefore preclude prosociality. On the contrary, prosociality appears to be a distinctive feature within LI. Children with LI tend to have problems across a range of social and behavioural measures. For example, using the same instrument, the SDQ, St Clair and colleagues (St. Clair, Pickles, Durkin, & Conti-Ramsden, 2011) found longitudinal evidence of hyperactivity, conduct problems, emotional difficulties and problems with peer relations during childhood and in adolescence in young people with LI. Data from the present investigation, indicate that prosociality is, in contrast, an area of relative strength, at least from early adolescence to young adulthood (and see also Lindsay & Dockrell, 2012; for broadly compatible findings in middle adolescence).

To our knowledge, this is the first study to use latent class analyses to examine age-related changes in levels of prosociality from early adolescence to young adulthood that includes a sample of young people with LI. Analyses revealed two different developmental trajectories for the LI group, which were stable and differed only in level of prosociality. Approximately one third of participants with LI in this study followed a moderate prosociality trajectory whilst the majority (71%) followed a prosocial trajectory. These findings corroborate previous longitudinal research. Kanacri and colleagues (e.g. Kanacri, Pastorelli, Eisenberg et al., 2014) found that the majority of the participants in their Italian sample were prosocial and their scores were close to the average for the scale used from age 13–21 years, albeit, this trajectory showing some quadratic variation across time. These investigators also found a low prosocial trajectory, which was not evident in this investigation. More variation in prosociality may be evident in studies like those of Kanacri and colleagues (Kanacri, Pastorelli, Eisenberg et al., 2014, Kanacri, Pastorelli, Zuffiano et al., 2014) which involved larger samples (over 500 participants).

There are two important points to note. First, all of the participants with LI had been identified as having language difficulties in childhood severe enough to warrant attending a specialist educational environment and not a mainstream classroom. All participants had thus received intensive intervention for their difficulties in language units attached to mainstream schools across England. All of the participants had continued to develop their expressive and receptive language skills during early adolescence to young adulthood (Conti-Ramsden et al., 2012). The early identification of language difficulties and the context of early, intensive language support received in educational contexts such as language units may have nurtured socialisation processes and the development of emphatic concern, which in turn influence the development of prosociality. Although it is likely that language units would have varied in their educational practice for inclusion (and access to non-affected peers), language units themselves afford opportunities for fostering prosociality, for example, helping others and working together. Lindsay and Dockrell (2012), for example, found more individual differences in prosociality in their sample of children with LI drawn from a variety of schools with different educational provision in two geographical areas (one city, one rural) in the UK. They found a higher proportion of children scoring in the “abnormal” range at one of the time points they studied (between 18 and 28% of children at 10, 12 and 16 years). The primary school years also appear to be a more vulnerable developmental period for children with LI. These children tend to be rated by their teachers as being less prosocial than their peers (Brinton et al., 2000, Fujiki et al., 1999, Hart et al., 2004). Future research that spans the primary as well as secondary school years would throw light as to potential developmental changes in prosociality in children and young people with LI.

Second, gender differences in prosociality confirmed previous research that prosocial behaviours are strongly associated with gender (Carlo et al., 2007, Kanacri et al., 2013, Nantel-Vivier et al., 2014). There were a significantly larger percentage of females in the prosocial trajectory as compared to the moderate prosociality trajectory. It is important to underline these findings, as it is not always the case that gender differences observed in the general population are also observed in individuals with developmental difficulties, such as LI. For example, with this same cohort Conti-Ramsden and Botting (2008) found that the usual gender difference in mental health in adolescence (where there is vulnerability for females) was not evident in young adulthood. Prosociality is different. Prosociality appears to be an area of relative strength in young people with LI and it follows the gender pattern observed in the general population.

### 4.2 Prosociality: higher levels of prosociality are protective in young adulthood

We found significant small to medium effects for social outcomes in young adulthood. Our data suggest that prosociality also acts as a protective factor in social functioning for young people with LI. The results indicated that a prosocial trajectory as compared to a moderate prosociality trajectory was associated with better community integration in young adulthood and was significantly protective against friendship difficulties for individuals with LI. Comparisons between individuals with LI in the prosocial trajectory and same-age peers also revealed significant differences in relation to friendship difficulties. However, these findings should be interpreted within the context that both individuals with LI in the prosocial trajectory and age-matched peers were close to floor on the measure of friendship difficulties (group friendship difficulties means ranging from 0.1for peers to 0.6 for LI on a 0–8 point scale). It should also be acknowledged that while we have identified an association between prosociality and better friendships and better community integration, the association analyses cannot determine causal relationships, nor the direction of causality. In fact, a case could be made that the causal direction is the reverse: that is, that these more favourable social circumstances nurture prosocial behaviour. Nevertheless, our data are very much in line with previous research with typical populations in studies which do point to protective effects (Carlo, Crockett, Wilkinson, & Beal, 2011; Cillessen et al., 2005; Markiewcz et al., 2001).

Prosociality, nonetheless, does not provide protection for all areas of functioning. In LI, associations of prosociality with behavioural functioning were weaker and non-significant. Comparisons with same age peers revealed individuals with LI exhibited significantly more aggressive behaviours in young adulthood regardless of their level of prosociality.

These data have important implications for fostering the strengths of young people with LI. Harnessing and further developing prosocial tendencies may lead to better social outcomes for young people with LI. We are not claiming that prosociality is the only factor impinging on friendships and community integration. The picture is complex and there are individual differences. For example, we know that a third of this same cohort experience problems with friendship in adolescence and young adulthood (Durkin and Conti-Ramsden, 2007, Mok et al., 2014, St. Clair et al., 2011). Nonetheless, a medium size effect size was observed between LI and AMP groups for friendships in this study, suggesting that in LI, being moderately prosocial may not be enough to confer protection, a higher “dosage” of prosociality is likely to be required.

To our knowledge, there has not been a systematic effort to build on the prosocial tendencies of individuals with LI in intervention programmes. It is more common to target areas of deficits rather than strengths. A good example is intervention research in autism. There is an abundance of programmes that target improving the social skills and prosocial behaviours of children and young people with autism spectrum disorders, although the effectiveness of such interventions has been limited (Bellini, Peters, Benner, & Hopf, 2007; Greenway, 2000).

In future work, it will be important to examine prosociality in young people with LI longitudinally from an earlier point in development and for research to include both intervention and observational designs. The inclusion of a broader array of measures of prosocial behaviours (e.g. experimental tasks and direct observations) is also needed. Although the SDQ prosocial scale has good reliability and has been used extensively in the literature, different measures are sensitive to different aspects of prosociality and their concurrent use may elucidate potential causal pathways to better outcomes for young people with LI.

## Conflicts of interest

The authors declare that they have no conflict of interest.

## Figures and Tables

**Table A1:** Correlations amongst predictor variables.

	1.	2.	3.	4.	5.	6.	7.	8.	9.	10.	11.	12.	13
1. Expressive Language T1	1												
2. Expressive Language T2	0.7^***^	1											
3. Expressive Language T3	0.8^***^	0.8^***^	1										
4. Receptive Language T1	0.6^***^	0.6^***^	0.6^***^	1									
5. Receptive Language T2	0.4^***^	0.6^***^	0.6^***^	0.5^***^	1								
6. Receptive Language T3	0.5^***^	0.6^***^	0.7^***^	0.5^***^	0.7^***^	1							
7. Performance IQ T1	0.3^**^	0.2^**^	0.4^***^	0.5^***^	0.4^***^	0.5^***^	1						
8. Performance IQ T2	0.2^*^	0.4^***^	0.4^***^	0.4^***^	0.6^***^	0.6^***^	0.8^***^	1					
9. Performance IQ T3	0.2^*^	0.4^***^	0.5^***^	0.5^***^	0.5^***^	0.6^***^	0.7^***^	0.8^***^	1				
10. Friendship Difficulties T3	−0.2	−0.3^***^	−0.3^***^	−0.4^**^	−0.3^***^	−0.3^***^	−0.1	−0.2^*^	−0.2^**^	1			
11. Community Integration T3	−0.3	0.1	0.1	−0.1	0.0	−0.1	−0.2	−0.1	−0.1	−0.2^*^	1		
12. Achenbach Aggressive T3	−0.2	−0.1	−0.2^*^	0.0	−0.1	−0.2^*^	−0.1	−0.2^*^	−0.3^**^	0.1	−0.2^*^	1	
13. Achenbach Rule Breaking T3	−0.0	−0.0	−0.1	0.0	−0.1	−0.0	0.0	0.0	−0.1	0.0	−0.2^**^	0.5^***^	1

^*^<.05, ^**^<.01, ^***^<.001. *Note*: variables at T1 are for LI sample only.

**Table 1:** Participants’ psycholinguistic profiles.

	Age 11 T1	Age 16 T2		Age 24 T3	
	LI (*n* *=* *130)*	LI (*n* *=* *126)*	AMP *(n* *=* *65)*	LI (*n* *=* *84*)	AMP (*n* *=* *64)*
Expressive Language	74.9 (12.3)	73.7(10.6)	98.9 (15.1)	70.6(15.6)	97.7(16.3)
Receptive Language	87.3(15.4)	83.9(17.1)	103.6(12.8)	83.5(18.6)	105.9(9.2)
Performance IQ	87.1 (23.4)	84.5(18.5)	104.0(14.8)	98.8(15.8)	113.2(10.8)

*Note*: AMP participants were enlisted from age 16.

**Table 2:** Model fit statistics for trajectory classes.

	Intercept Only				Linear				Quadratic	
	LI		AMP		LI		AMP		LI	
	Adj. AIC	BIC	Adj. AIC	BIC	Adj. AIC	BIC	Adj. AIC	BIC	Adj. AIC	BIC
1 class solution	1288.05	1309.88	**411.11**	**424.36**	1289.17	1313.56	413.59	428.41	1290.14	1317.06
2 class solution	**1279.92**	**1306.84**	413.63	429.93	1276.71	1308.47	415.19	434.13	1275.51	1312.14
3 class solution	1284.73	1316.59	418.95	437.88	1284.15	1323.10	423.55	445.59	1281.85	1327.50

*Note*: The chosen models are shown in bold.

**Table 3:** Prosocial scores for each of the classes.

	Moderate Prosociality LI	Prosocial LI	AMP
Age 11 (T1)	5.6(2.0) *(n* *=* *37)*	8.9(1.5) *(n* *=* *88)*	–
Age 16 (T2)	6.2(1.9) *(n* *=* *38)*	8.4(1.4) *(n* *=* *92)*	8.8(1.3) *(n* *=* *65)*
Age 24 (T3)	6.2(2.1) *(n* *=* *21)*	8.4(1.4) *(n* *=* *59)*	8.6(1.5) *(n* *=* *65)*

Values are mean (SD).

**Table 4:** Outcome comparisons for trajectory classes.

Outcome	Means (SD)			One-way ANOVA	
	Moderate Prosociality LI	Prosocial LI	AMP	F *df* (2,142)	Adjusted R^2^
Friendship Difficulties *(n* *=* *147)*	2.3(2.7)^a^	0.6(1.1)^b^	0.1(0.4)^c^	23.65^***^	.24
Community Integration *(n* *=* *145)*	36.7(7.6)^a^	40.5(6.7)^b^	41.9(6.2)^b^	4.66^*^	.05
					
Achenbach Aggressive *(n* *=* *145)*	6.9(6.9)^a^	5.9(5.1)^a^	4.1(3.8)^b^	3.58^*^	.04
Achenbach Rule Breaking *(n* *=* *145)*	3.0(3.2)^a^	2.3(2.3)^a^	2.3(2.9)^a^	.54	.01

^*^<.05, ^**^<.01, ^***^<.001. *Note*: Means within rows not sharing a superscript are significantly different, *p* < .05.

## Appendix A

See Table A1.
